# Multi-Omics Integration Unravels the Genetic and Hormonal Regulatory Mechanisms Underlying Increased Main Stem Node Number in Soybean

**DOI:** 10.3390/plants15101418

**Published:** 2026-05-07

**Authors:** Jinbo Zhang, Yongbin Wang, Weiwei Tan, Bixian Zhang, Chunxu Leng, Yang Peng, Licheng Wu, Yuanhang Zhou, Aoran Song, Zhaojun Liu

**Affiliations:** 1 Biotechnology Research Institute, Heilongjiang Academy of Agricultural Sciences, Harbin 150028, China; zjb19881210@163.com (J.Z.); wyby119@126.com (Y.W.); weiwei20098@yeah.net (W.T.); hljsnkyzbx@163.com (B.Z.); lengchx@haas.cn (C.L.); yapybb@163.com (Y.P.); yezi_320@163.com (L.W.); 13504551002@163.com (Y.Z.); songar105@163.com (A.S.); 2 Heilongjiang Laboratory of Crop and Livestock Molecular Breeding, Harbin 150028, China

**Keywords:** BSA-seq, soybean, transcriptome, main stem node number, *GmGASA32*

## Abstract

Soybean (*Glycine max* L.) yield is critically influenced by the number of nodes on the main stem (MSN), which serves as the primary site for pods and seeds. To elucidate the genetic mechanisms underlying MSN, we conducted a multi-omics analysis integrating bulk segregant analysis sequencing (BSA-seq), phytohormone, and transcriptome profilings in a soybean mutant, LSD914, which exhibits a significantly increased MSN number compared to its wild-type parent, HN48. BSA-seq of an F2 population identified 27 candidate genomic regions spanning 2.92 Mb, primarily on chromosome 18. Within these regions, 149 genes harbored non-synonymous SNPs and 26 genes contained frameshift InDels, with functional enrichment pointing to pathways in plant hormone signal transduction and developmental regulation. Phytohormone profiling revealed a distinct shift in LSD914, characterized by down-regulation of jasmonates, salicylates, and auxins, alongside specific accumulation of cis-zeatin. Integrative transcriptome analysis identified *Glyma.18G259400*, a gene encoding a gibberellin-regulated protein (GmGASA32), which was consistently and significantly down-regulated in LSD914 across all developmental stages and tissues. This finding contrasts with previous reports of its overexpression promoting plant height, suggesting a nuanced, context-dependent regulatory role. Our integrated approach identifies a key set of candidate genes and highlights *GmGASA32* as a pivotal node in a hormone signaling network that orchestrates soybean node number, providing valuable targets for breeding high-yield soybean varieties with optimized plant architecture.

## 1. Introduction

Soybean (*Glycine max* L.) is a cornerstone of global agriculture, serving as a critical source of protein and oil for human consumption and animal feed [1]. As a typical legume, its yield is intricately linked to its architectural structure; leaves, flowers, and pods are all borne at the nodes of the stem [2]. Therefore, the number of nodes on the main stem (MSN) is a fundamental agronomic trait that directly dictates the potential number of pods and seeds per plant, ultimately influencing grain yield [3]. In China, the persistent challenge of low soybean yield per unit area, coupled with high import dependency, underscores the urgent need to dissect and leverage the genetic components of yield-related traits like MSN [1,4]. Optimizing node number is considered a key strategy for breaking the yield bottleneck, particularly under dense planting conditions where a balanced increase in nodes can enhance pod count without exacerbating lodging risk [5,6,7].

The regulation of soybean node number is a complex process involving multiple layers of genetic and environmental control. Early genetic studies established MSN as a quantitative trait with high heritability, enabling effective selection in early generations [8,9,10]. Subsequent mapping efforts using various populations, including recombinant inbred lines (RILs) and chromosome segment substitution lines (CSSLs), have identified numerous quantitative trait loci (QTLs) within the soybean genome [11,12]. These efforts have revealed that node number is controlled by both major-effect genes and minor-effect polygenes, with significant contributions from maternal effects and transgressive segregation [13,14,15]. Although previous QTL mapping using RILs and CSSLs has identified over 50 MSN-related loci across multiple linkage groups, such as D1a, D1b, N, C1, and A1 [8,16,17], most of these QTLs were defined using SSR markers with large confidence intervals. Moreover, due to the paleotetraploid nature of the soybean genome, these QTLs have rarely been stably validated across different environments or genetic backgrounds, greatly hindering the cloning of key causal genes and their application in breeding.

Notably, several key soybean genes exhibit pleiotropy. For instance, Dt1 influences stem growth habit, flowering time, and node number [18,19], while Tof18 and AP1 homologs modulate flowering time along with main stem node number [20,21]. The Dt2 gene exemplifies pleiotropic regulation, acting through protein–protein interactions to simultaneously influence branching and node number, highlighting the interconnected nature of plant architecture [22]. These findings imply that the molecular network controlling node number is highly coupled with flowering and plant height pathways. Beyond genetic factors, environmental signals such as photoperiod and temperature exert a profound influence. As a typical short-day plant, soybean’s node formation is tightly coupled to its developmental timeline; long days delay flowering, prolonging the vegetative phase and leading to more nodes, while short days accelerate maturation, reducing node number [23,24]. Similarly, temperature modulates the rate of development, with optimal ranges promoting node differentiation, while extremes can shorten the growth window [25,26].

At the molecular level, the formation of nodes is orchestrated by a sophisticated network of phytohormones and transcription factors. Gibberellin (GA) is a central regulator, where the RIN1 gene controls internode elongation by modulating the degradation of STF proteins, which in turn influences the expression of GA deactivation genes, thereby affecting active GA levels and subsequent node development [7]. Other hormones, including auxin (IAA), cytokinin (CTK), and brassinosteroids (BR), also play critical roles, with cytokinins promoting cell division to provide the cellular foundation for new node initiation [27,28]. At the transcriptional level, various transcription factor families, such as MYB, MADS-box, and AP2/ERF, integrate these hormone signals to precisely control the expression of downstream genes involved in node differentiation [29,30]. Despite these advances, the coordinated action of these pathways in determining the final node number remains incompletely understood.

Recent advances in high-throughput sequencing have enabled powerful methods like bulk segregant analysis sequencing (BSA-seq) to rapidly map genomic regions associated with complex traits [31]. When combined with transcriptome sequencing (RNA-seq), this approach allows for the efficient identification of candidate genes that not only reside within key genomic intervals but also exhibit differential expression that correlates with the phenotype of interest [32,33,34]. In this study, we employed this integrated multi-omics strategy, combined with phytohormone profiling, to dissect the genetic architecture of MSN in a stable multi-node soybean mutant, LSD914. Our goal was to identify key regulatory genes and pathways responsible for the increased node number phenotype, providing valuable insights and molecular targets for breeding programs aimed at developing high-yielding soybean varieties with optimized plant architecture.

## 2. Results

### 2.1. Phenotypic Evaluations of Parental and Segregating Population

**Figure 1:**
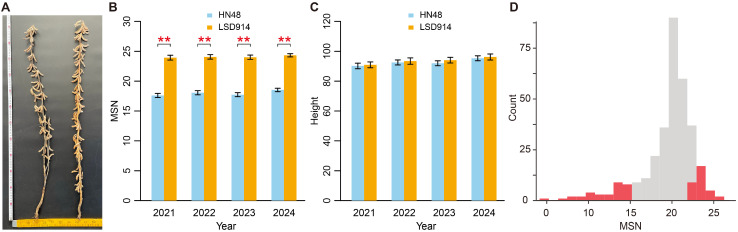
Phenotypic characterization of soybean varieties HN48 (wild-type) and LSD914 (multi-node mutant). (**A**) Representative photographs of HN48 (left) and LSD914 (right) at maturity. Scale bar = 10 cm. (**B**) Annual changes in main stem node (MSN) number from 2021 to 2024. Data are shown as mean ± SD (*n* = 15 plants per line per year, three biological replicates). ** *p* < 1 × 10^−5^ (Wilcoxon rank-sum test). (**C**) Annual changes in plant height for the same lines (same statistics). (**D**) Schematic of phenotypic bulks for BSA-seq. A total of 322 F2 individuals were phenotyped; two extreme bulks with red color (MSN-M for more nodes, MSN-F for fewer nodes) were each composed of 32 individuals from the tails of the distribution. Parental controls (HN48-CK and LSD914-CK) were also included.

In this study, we investigated the genetic mechanisms associated with the main stem node (MSN) number. A natural population comprising two lines, the wild-type Heinong 48 (HN48) and multi-node mutant LSD914, was evaluated from 2021 to 2024 (Figure 1A–C). Although plant heights were comparable (~90–96 cm) between the two lines, their MSN numbers differed significantly across the four years (Figure 1B,C; Wilcox rank sum test, *p* < 1 × 10^−5^). Specifically, HN48 averaged 17.98 nodes, while LSD914 reached 24.08. LSD914 displayed ~34% more nodes without height increase. Because total plant height remained similar, the increased node number in LSD914 is necessarily accompanied by a proportional reduction in average internode length. This indicates that the LSD914 mutation promotes node initiation while suppressing internode elongation, resulting in a compact, high-node architecture. This genetically stable trait makes these lines ideal for BSA-seq analysis of MSN phenotype.

An F2 segregating population was developed for BAS-seq, including 322 accessions for phenotypic evaluations. Two extreme bulks were constructed: a high node number bulk (MSN-M) and a low node number bulk (MSN-F), each of which consisted of 33 individuals with the most extreme phenotypes (Figure 1D). Additionally, the two parental lines, HN48-CK and LSD914-CK, were also included in the subsequent analysis.

### 2.2. BSA-Seq Data Analysis and Variant Detection

To efficiently map the genomic regions controlling the MSN trait, bulk segregant analysis sequencing (BSA-seq) was performed on the two parental lines and the two extreme bulks. A total of approximately 29.5 Gb of clean data were generated from the four samples (Table S1). The mapping rates ranged from 98.61% to 99.19%, and the average coverage depth for the two bulks was approximately 30×, ensuring robust variant detection (Table S1).

Following read alignment and variant calling, a total of 1,962,596 single-nucleotide polymorphisms (SNPs) and 448,060 small insertions/deletions (InDels) were identified across all samples (Figures S1 and S2; Table S2). After stringent filtering on the variants between the two extreme bulks, 750,802 SNPs and 163,994 InDels were retained for subsequent association analysis.

### 2.3. Identification of Candidate Genomic Regions

Two complementary methods, the Δ(SNP-index) (or Δ(InDel-index)) method and the Euclidean Distance (ED) method, were employed to identify genomic regions statistically associated with the target trait of MSN number. The Δ-index method calculates the allele frequency difference between the two bulks, while the ED method measures the genetic distance between them.

**Figure 2:**
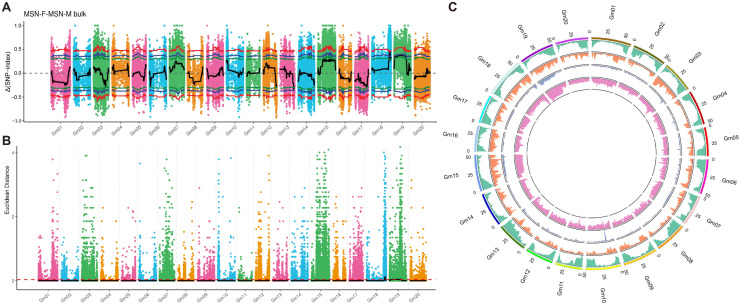
Genome-wide identification of candidate genomic regions associated with MSN number using two analytical methods and SNP variants. (**A**) Δ(SNP-index) analysis. The Δ(SNP-index) value (difference in SNP-index between the high-node and low-node bulks) is plotted for each SNP across the 20 soybean chromosomes. The upper and lower dashed lines indicate the 95% confidence intervals (permutation test, 1000 iterations). Colored dots represent the calculated SNP-index (or ΔSNP-index) values, and the black line represents the fitted SNP-index (or ΔSNP-index) values. The bottom figure shows the distribution of ΔSNP-index values. The red line indicates the significance threshold at the 0.99 confidence level, the blue line at the 0.95 confidence level, and the green line at the 0.90 confidence level. (**B**) Euclidean distance (ED) analysis based on the same SNP dataset. ED values were calculated using a sliding window (window size = 1 Mb, step = 100 kb). The red dashed line represents the significance threshold (median + 3 × SD). Colored dots indicate the ED value of each SNP site, while the black line represents the fitted ED value. The red dashed line indicates the significance threshold for association. (**C**) Overlap of candidate regions identified by both Δ-index and ED methods using SNPs. From outer to inner: chromosome coordinates; gene distribution; SNP density distribution; SNP-ED value distribution; ΔSNP-index value distribution.

For SNPs, the Δ(SNP-index) analysis revealed 66 candidate regions, spanning a total of 3.70 Mb (Figure 2A). The ED analysis identified 45 candidate regions, covering 27.65 Mb (Figure 2B). For InDels, the Δ(InDel-index) method yielded 13 candidate regions (3.22 Mb), while the ED method identified 2 regions (14.04 Mb) (Figure S3A,B). Finally, the intersection of all the results, including two methods and two variation types, produced 27 candidate regions totaling 2.92 Mb (Tables S3 and S4). These significant regions were observed in two chromosomes, chr18 and chr19 (Figure 2C; Table S5). Of them, the largest genomic region was located at chr18, spanning 2.84 Mb, accounting for 97.26% of the total significant regions, which might play the most important role in the genetic mechanisms of MSN numbers.

### 2.4. Functional Annotation of Genes Within Candidate Regions

A total of 355 genes were annotated within the final set of 27 candidate regions, of which 341 possessed functional annotations (Table S6). To prioritize candidate genes most likely involved in the regulation of the MSN trait, we focused on variants with predicted high functional impacts. Analysis of the SNPs within the 27 candidate regions identified 477 non-synonymous mutations between the two parents (HN48-CK vs. LSD914-CK) and 95 non-synonymous mutations between the two bulks (MSN-F vs. MSN-M). For InDels, 36 frameshift mutations were detected between the parents, and 15 were detected between the bulks. Finally, we identified 149 genes harboring these non-synonymous mutations and 26 genes with frameshift mutations, which were considered primary candidates (Tables S7 and S8).

**Figure 3:**
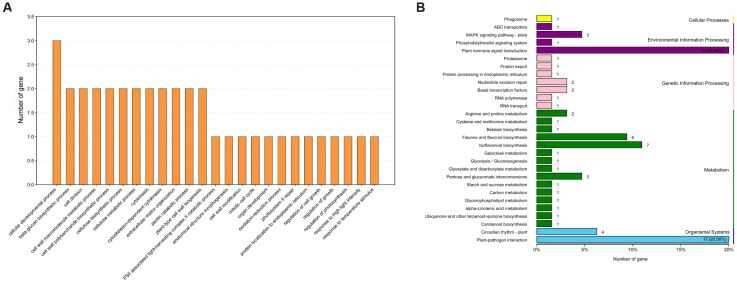
Functional enrichment analysis of candidate genes (*n* = 355) located within the 27 BSA-seq-derived candidate regions. (**A**) Gene Ontology (GO) enrichment of biological processes. Only terms with adjusted *p*-value < 0.05 (Benjamini–Hochberg FDR) are shown. The *x*-axis represents the fold enrichment, and the dot size indicates the number of significant genes. (**B**) KEGG pathway enrichment for the same gene set. The number of gene are presented in each KEGG pathway.

Gene Ontology (GO) enrichment analysis, which passed the significance threshold (adjusted *p* < 0.05), revealed significant enrichment in pathways critical for growth and development (Figure 3A; Table S9). Notably, a number of GO categories of biological processes associated with development were significantly over-represented, such as cell division (GO:0051301), cellulose metabolic process (GO:0030243), extracellular matrix organization (GO:0030198), regulation of photosynthesis (GO:0010109), and regulation of growth (GO:0040008). In contrast, Kyoto Encyclopedia of Genes and Genomes (KEGG) pathway analysis identified several related to plant hormone signal transduction and carbohydrate metabolism, which are known to be involved in developmental regulation (Figure 3B; Table S10). The pathways of plant hormone signal transduction (ko04075) and plant-pathogen interaction (ko04626) possessed the most primary candidate genes. Collectively, the functional annotation suggests that the candidate genes may influence node number by modulating the developmental processes and hormone signaling pathways during growth and development.

### 2.5. Phytohormone Profiling in Parental Lines

**Figure 4:**
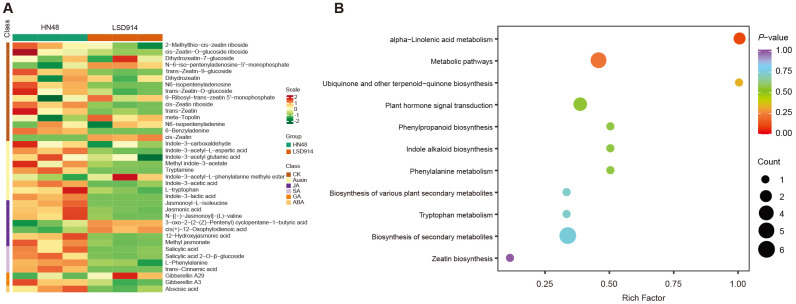
Phytohormone profiling of HN48 and LSD914 parental lines (*n* = 3 biological replicates per line). (**A**) Heatmap of 38 detected phytohormone-related metabolites. Color represents *p*-value (red: low, purple: high). Fourteen metabolites showed significant differential accumulation (|log_2_ fold change| ≥ 1, *p* < 0.05, *t*-test). Jasmonates (JA, MEJA, JA-Ile, JA-Val, 12-OH-JA), salicylates (SA, SAG), and auxins (IAA-Asp, TRA, ILA) were down-regulated in LSD914, while cis-zeatin (cZ) was detected only in LSD914. (**B**) KEGG enrichment of the 14 differential metabolites. The only up-regulated metabolite (cZ) is annotated in the metabolic pathways, secondary metabolism, and zeatin biosynthesis pathways.

To investigate the potential link between candidate genes and phytohormone regulation, a targeted metabolomics analysis was performed to quantify 108 phytohormone-related metabolites in the two parental lines, HN48 and LSD914 (Table S11). Of these, 38 metabolites were detected in either of the two lines (Figure 4A). Among the detected metabolites, 14 exhibited significant differential accumulation between the two lines (Table S12). Notably, the majority of differentially accumulated metabolites were down-regulated in LSD914 compared to HN48, with only one compound showing up-regulation.

Jasmonate (JA) pathway metabolites, including jasmonic acid (JA), methyl jasmonate (MEJA), jasmonoyl-L-isoleucine (JA-ILE), N-[(+)-Jasmonoyl]-L-valine (JA-Val), and 12-hydroxyjasmonic acid (12-OH-JA), showed pronounced reduction in LSD914 (Figure 4A; Table S12). Salicylic acid (SA) and its glucoside conjugate (SAG) were also substantially reduced in LSD914, with fold changes of 0.49 and 0.33, respectively. Several auxin-related metabolites exhibited similar patterns, including indole-3-acetyl-L-aspartic acid (IAA-Asp), tryptamine (TRA), and indole-3-lactic acid (ILA), all of which were markedly decreased or even undetectable in LSD914. In contrast to the other two cytokinins (CK, trans-Zeatin-9-glucoside and 6-Benzyladenine), the cis-zeatin (cZ) was exclusively detected in LSD914, while being absent in HN48 (Figure 4A; Table S12).

KEGG analysis of these differential metabolites revealed four over-represented pathways, including plant hormone signal transduction (ko04075), alpha-linolenic acid metabolism (ko00592), metabolic pathways (ko01100), and biosynthesis of secondary metabolites (ko01110) (Figure 4B). As the only up-regulated phytohormone in LSD914, cZ was presented in the two KEGG pathways of metabolic pathways (ko01100), biosynthesis of secondary metabolites (ko01110), and zeatin biosynthesis (ko00908) (Figure 4B). Especially, the cZ was the only up-regulated one in the former two pathways associated with metabolisms, while all of the other phytohormones were observed to be down-regulated. Collectively, the observed phytohormone profiles, characterized by widespread down-regulation of jasmonates, salicylates, and auxins coupled with specific accumulation of cis-zeatin in LSD914, are suggestive of the physiological mechanisms of MSN number by modulating multiple hormone biosynthesis and signaling pathways.

### 2.6. Transcriptome Assembly and Quality Assessment

To explore the transcriptional dynamics underlying the phenotypic differences between LSD914 and HN48, we performed RNA-seq analysis on 36 samples derived from leaf and stem tissues at three developmental stages: V2 (seedling), R2 (full bloom), and R4 (full pod). Mapping statistics were highly robust across all 36 sample groups. On average, 93.62% of reads were uniquely mapped (range: 91.71–94.91%). Notably, a comprehensive reference transcript dataset was constructed using StringTie and RTDmaker, serving as the basis for transcript abundance quantification with Salmon. Finally, we obtained 53,627 genes and 200,419 transcripts for further analyses based on expression quantities.

Principal component analysis revealed clear clustering of samples at the gene level, with distinct separation by tissue type (leaf vs. stem) and developmental stage (V2, R2, R4) (Figure 4A). Biological replicates clustered tightly, indicating high reproducibility. Transcript-level PCA showed similar but less distinct grouping, reflecting additional isoform-level complexity (Figure 4B).

### 2.7. Identification of Differentially Expressed Genes

**Figure 5:**
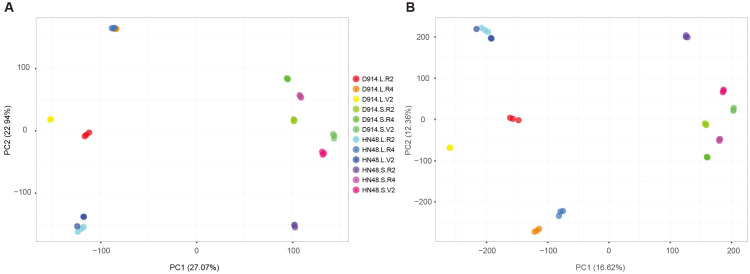
Principal component analysis (PCA) of transcriptome profiles from 36 samples (leaf and stem tissues at V2, R2, R4 stages; 3 biological replicates per genotype-tissue-stage combination). (**A**) Gene-level PCA. PC1 (62.3% variance) separates tissue types (leaf: L; stem: S); PC2 (18.7% variance) separates developmental stages (V2, R2, R4). (**B**) Transcript-level (isoform-level) PCA. Replicates cluster similarly, but with less separation between stages, indicating isoform-level complexity.

To delineate transcriptional differences between LSD914 and HN48, we performed a comparative analysis. A total of six pairwise comparisons were conducted between the two genotypes across each tissue-stage combination: LSD914.L.V2 vs. HN48.L.V2, LSD914.L.R2 vs. HN48.L.R2, LSD914.L.R4 vs. HN48.L.R4, LSD914.S.V2 vs. HN48.S.V2, LSD914.S.R2 vs. HN48.S.R2, and LSD914.S.R4 vs. HN48.S.R4 (Figure 5; Figures S4 and S5). The number of differentially expressed genes (DEGs) varied substantially across tissues and stages, ranging from 3325 to 11,552 (Table S13). In leaf tissue, the V2 stage exhibited the largest number of DE genes (11,470), followed by R2 (8222) and R4 (6513). In stem tissue, the R2 stage showed the highest number of DE genes (11,552), while the R4 stage exhibited the fewest (3325). The distribution of up- and down-regulated genes was also asymmetric across comparisons. These results indicate that the transcriptional response between LSD914 and HN48 is both tissue- and stage-specific.

**Figure 6:**
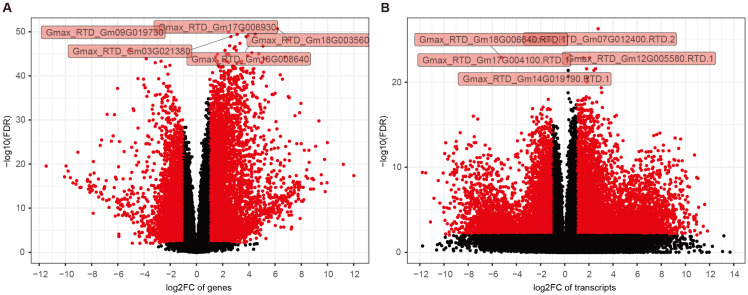
Volcano plots showing differential expression between LSD914 and HN48 in leaf tissue at the R2 stage (L.R2). (**A**) Gene-level volcano plot. Each point represents a gene: red for DEGs and black for non-DEGs. The horizontal dashed line indicates adjusted *p*-value = 0.01 (Benjamini–Hochberg); vertical dashed lines indicate log_2_ fold change = ±1. Five DEGs have been presented, including *Gmax_RTD_Gm09G019730*, *Gmax_RTD_Gm03G021380*, *Gmax_RTD_Gm17G006930*, *Gmax_RTD_Gm18G003560*, and *Gmax_RTD_Gm16G008640*. (**B**) Transcript-level (isoform-level) volcano plot. Five DTGs have been presented, including *Gmax_RTD_Gm14G019190.RTD.1*, *Gmax_RTD_Gm18G006640.RTD.1*, *Gmax_RTD_Gm07G012400.RTD.2*, *Gmax_RTD_Gm17G004100.RTD.1*, and *Gmax_RTD_Gm12G005580.RTD.1*. Their functional annotations are based on GO and KEGG analyses (see Materials and Methods).

Beyond changes in overall gene expression, we investigated transcriptome-wide alterations at the transcript level (Table S13). Our analysis detected pervasive post-transcriptional regulation across all comparisons, with the number of differentially expressed transcripts (DETs) ranging from 4698 to 22,245 (Table S13). Consistent with the DE analysis, the largest number of DETs was found in the leaf tissue and V2 stage, while the stem tissue possesses the most DEGs and DETs at the R2 stage. This indicates that the V2 stage (seedling) of leaf and the R2 stage (full bloom) of stem serve as hubs not only for differential gene expression but also for significant splicing regulation and transcript isoform switching, suggesting a complex layer of transcriptional control accompanying developmental transitions (Figure 6).

### 2.8. Integrated Analysis of BSA-Seq Candidates and Differential Expressions

**Figure 7:**
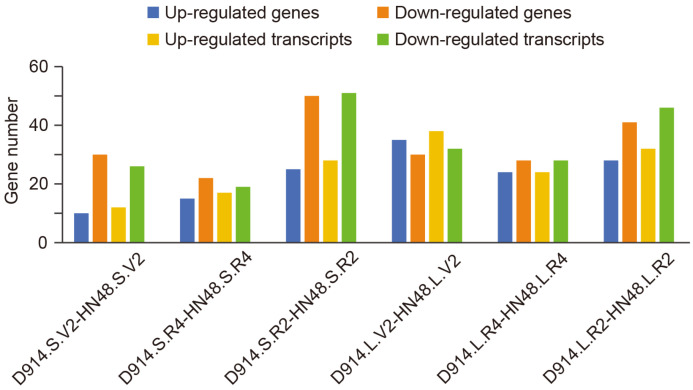
Numbers of differentially expressed genes (DEGs) and transcripts (DETs) that overlap with the BSA-seq candidate regions, shown for six tissue-stage comparisons between LSD914 and HN48. For each comparison, up-regulated (blue) and down-regulated (orange) genes are plotted separately. The full list of overlaps is provided in Supplementary Tables S14 and S15.

According to the integrated analysis of BSA-seq candidate genes and multi-level transcriptomic data across six comparative groups, the overlap with candidate genomic regions was characterized at both gene and transcript levels (Tables S14 and S15). The results showed that the number of significantly up-regulated and down-regulated genes varied among the groups, with down-regulated genes generally outnumbering up-regulated ones in most comparisons (Figure 7). In the LSD914.S.V2-HN48.S.V2 group, 10 up-regulated and 30 down-regulated genes were identified; in LSD914.S.R4-HN48.S.R4, 15 up-regulated and 22 down-regulated; in LSD914.S.R2-HN48.S.R2, 25 up-regulated and 50 down-regulated; in LSD914.L.V2-HN48.L.V2, 35 up-regulated and 30 down-regulated; in LSD914.L.R4-HN48.L.R4, 24 up-regulated and 28 down-regulated; and in LSD914.L.R2-HN48.L.R2, 28 up-regulated and 41 down-regulated. At the transcript level, similar trends were observed: the highest numbers of differentially expressed transcripts were also found in LSD914.S.R2-HN48.S.R2 and LSD914.L.R2-HN48.L.R2.

Remarkably, three genes were consistently differentially expressed across all six groups, all showing down-regulated patterns in the LSD914 line, including *Glyma.18G259400*, *Glyma.18G245400*, and *Glyma.18G245500*. At the transcript level, four transcripts were consistently down-regulated across all six contrasts in the LSD914 line, including the *Glyma.18G259400* (Gibberellin-regulated protein), *Glyma.18G249800* (Subtilisin-like protease), *Glyma.18G266200* (Histone H3), and *Glyma.18G269800* (Uncharacterized protein). The identification of transcript-specific regulation not captured at the gene level highlights the added resolution of alternative splicing analysis in revealing functional isoforms. The consistent down-regulation of these genes and transcripts across diverse conditions suggests they may play fundamental roles in response to experimental treatments and represent high-confidence candidates for further functional validation. Interestingly, the gene *Glyma.18G259400*, differentially expressed at both gene and transcript level, was annotated as *GASA32* (gibberellin-regulated protein 32), involved in Gibberellin-related hormone signaling. Fourteen potential QTLs were localized to the GmGASA32-containing region, suggesting that this region may influence expression patterns and contribute to the MSN difference in LSD914 (Table S16). However, whether *GmGASA32* itself is the causal gene or is merely linked to the causative mutation requires functional validation.

## 3. Discussion

In this study, we employed an integrated multi-omics strategy—combining BSA-seq, transcriptome sequencing, and phytohormone profiling—to dissect the genetic architecture of main stem node (MSN) number in a soybean mutant with a high-node phenotype. The power of this integrated approach lies in its ability to converge on high-confidence candidates by simultaneously considering genomic location, functional variation, and expression dynamics. BSA-seq provided an unbiased genome-wide scan, pinpointing 27 candidate regions totaling 2.92 Mb on chromosomes 18 and 19. The subsequent transcriptomic analysis across multiple tissues and developmental stages added a critical functional layer, allowing us to identify genes within these regions that were consistently differentially expressed in the mutant. This combined strategy is more effective than either method alone, as it filters out positional candidates that are not transcriptionally responsive, thereby significantly narrowing the list of genes for functional validation [35,36]. By narrowing the candidate regions to 2.92 Mb and, more importantly, adding functional validation through transcriptomic and phytohormone profiling, we identified a core regulatory gene from a pool of positional candidates—a level of resolution that traditional mapping methods could not achieve.

A key feature of the LSD914 phenotype is the uncoupling of node number from plant height: more nodes are produced without an increase in total stem length, implying shorter internodes. Thus, the underlying genetic and hormonal changes likely affect both node initiation (positively) and internode elongation (negatively), or they shift the balance between these two processes. Among the prioritized candidates, *Glyma.18G259400*, encoding the gibberellin-regulated protein GmGASA32, emerged as a central node. Our data consistently showed its down-regulation in LSD914 across all contrasts, which is particularly intriguing given a previous report that its overexpression promotes plant height [37]. This apparent discrepancy suggests a context-dependent regulatory function. We hypothesize that in the LSD914 mutant, the down-regulation of *GmGASA32* may act as a developmental switch, repressing internode elongation—a function consistent with GASA proteins acting as negative regulators in some contexts [38]—while simultaneously promoting node initiation. This model is strongly supported by our phytohormone profiling, which revealed a unique landscape in LSD914, characterized by the down-regulation of auxins, jasmonates, and salicylates, alongside a specific accumulation of the cytokinin cis-zeatin (cZ). Cytokinins, particularly cZ, are known to promote cell division and could drive the formation of additional nodes. Therefore, we propose that *GmGASA32* may function as an integrator of these hormone signals, potentially mediating the crosstalk between the GA and CK pathways to balance the trade-off between internode elongation and node number. Recent advances in auxin signaling biology have highlighted the dynamic nature of auxin biodynamics and its integral role in coordinating plant growth and development through complex crosstalk with other phytohormones [39]. This framework supports our observation that reduced auxin-related metabolites in LSD914 may contribute to a shift in hormone balance, favoring node initiation over internode elongation. Collectively, the reduction in auxins, JA, and SA may lower the threshold for node initiation by attenuating signals that typically promote elongation and stress-related growth suppression. The concurrent accumulation of cZ, a cytokinin known to stimulate cell division in shoot meristems, likely provides a localized positive cue for new node formation. Thus, the hormone landscape in LSD914 appears to shift from an ‘elongation-dominant’ state (higher auxins, JA, SA) toward a ‘node-initiation-permissive’ state (elevated cZ, reduced counteracting hormones). However, the specific accumulation of cis-zeatin (cZ) in LSD914 is a striking observation, but our data do not distinguish whether elevated cZ drives node initiation or is a secondary consequence of altered shoot architecture. Determining causality would require exogenous cZ application, tissue-specific cZ measurement during early node development, or genetic manipulation of cZ biosynthetic genes.

Identifying *GmGASA32* as a key regulator of MSN also provides a new perspective on how pleiotropic genes balance different developmental processes in soybean. Unlike Dt1 or Tof18, which broadly affect flowering, plant height, and node number [19,20,21], the differential expression of *GmGASA32* in LSD914 did not significantly alter flowering time or plant height (Figure 1A), suggesting that its regulatory path is more specific, mainly mediating the trade-off between internode elongation and node initiation. This makes *GmGASA32* an ideal target for precise editing to optimize plant architecture while avoiding undesirable pleiotropic effects. This refined perspective positions *GmGASA32* as a key regulator of plant architecture beyond a simple growth promoter.

Beyond *GmGASA32*, our integrated analysis identified several other promising candidates that warrant further investigation. Genes such as *Glyma.18G245400* and *Glyma.18G245500* were also consistently down-regulated in LSD914, alongside transcripts for a subtilisin-like protease and a histone H3 variant. Subtilisin-like proteases are implicated in plant development and signaling, while histone variants are central to epigenetic regulation of gene expression. The consistent differential expression of these genes suggests they may form part of a regulatory module controlling node number, potentially acting upstream or downstream of *GmGASA32*. Their roles in this process remain unexplored and present exciting avenues for future functional characterization.

The identification of *GmGASA32* and its associated genomic region (chr18) has significant implications for soybean breeding. The localization of 14 potential QTLs within this region underscores its importance as a genetic hotspot for node number variation. The consistent down-regulation pattern observed in LSD914 provides a clear expression-based marker for this favorable allele. This information can be directly leveraged for marker-assisted selection (MAS) to introgress the high-node trait into elite varieties. Furthermore, the understanding of *GmGASA32* as a regulatory node offers a precise target for gene editing technologies. By modulating its expression or its interaction with partners like *GmCDC25*, we may be able to uncouple the positive correlation between node number and plant height, a critical challenge for achieving high yields under dense planting conditions. In summary, the integrated analysis presented here not only elucidates a key molecular mechanism underlying node number variation but also delivers tangible targets for the genetic improvement of soybean plant architecture and yield.

It should be emphasized that, despite the consistent differential expression and genomic localization of *GmGASA32*, we cannot rule out the possibility that it is linked to rather than directly responsible for the causal mutation. *GmGASA32* is presented as a high-priority candidate that requires functional validation (e.g., gene editing or complementation) to establish causality. Thus, the inferred role of *GmGASA32* in regulating node number remains correlative and requires direct experimental confirmation. Additionally, our analyses were performed using two soybean varieties (HN48 and LSD914) grown under field conditions in a single geographic location. Broader validation of the *GmGASA32*-associated QTLs across diverse genetic backgrounds and environments is a logical next step for soybean breeding. Finally, while our bulk transcriptome analysis captured average expression changes across whole tissues, emerging technologies such as single-cell and spatial transcriptomics could, in the future, resolve cell-type-specific regulatory programs within the shoot apical meristem, potentially uncovering the precise spatial domains where *GmGASA32* and hormone signals operate [40].

## 4. Materials and Methods

### 4.1. Plant Materials and Phenotyping

The soybean (*Glycine max*) parental line HN48 and its induced mutant line LSD914, which exhibits a significant increase in main stem node (MSN) number, were used in this study. The mutant line has been selfed to the M7 generation and showed stable phenotypic performance. Field trials were conducted over four consecutive years (2021–2024), with node number and plant height recorded for both lines. Growth criteria included plant height (cm), main stem node number, branch number, internode length (cm), days to flowering (R1), days to maturity (R8), pod number per plant, and 100-seed weight (g). All measurements were taken at maturity (R8) over four years (2021–2024). Fifteen plants per line were randomly selected for phenotyping each year, with three biological replicates. Statistical analysis was performed using a non-parametric test (Wilcox rank sum test) to confirm the significance of phenotypic differences.

### 4.2. Construction and Sequencing for Bulk Segregant Analysis by Sequencing (BSA-Seq)

An F2 population of soybean (*Glycine max*) segregating, derived from a cross between line HN48 and LSD914, for the node number trait (fewer versus more nodes of the main stem) was developed for BSA-seq. F2 individuals were sampled at the V2 stage (third trifoliate leaf). Young leaves were collected individually, immediately frozen in liquid nitrogen, and stored at −80 °C. After maturity phenotyping, 32 individuals with extremely high-node and 32 with extremely low-node phenotypes were selected, and equal amounts of leaf tissue were pooled to form the two bulks for DNA extraction and sequencing, including the “Fewer main stem node number” bulk (MSN-F) and the “More main stem node number” bulk (MSN-M). The two parental lines, HN48-CK and LSD914-CK, were similarly included. High-quality genomic DNA from the two parental lines and the two phenotypic bulks was fragmented, and sequencing libraries were prepared following the standard Illumina TruSeq protocol. The libraries were subjected to paired-end (2 × 150 bp) sequencing on an Illumina platform.

### 4.3. Data Processing and Variant Calling

Raw sequencing reads were processed to remove adapter sequences and low-quality bases using fastp (v0.23.1) [41]. The resulting clean reads for all four samples were independently aligned to the soybean reference genome (http://phytozome.jgi.doe.gov/; *Glycine max* Wm82.a4.v1; accessed on 1 February 2026) using BWA-MEM (v2.2) [42]. Subsequent processing, including sorting and duplicate marking, was performed with SAMtools (v1.9) [43] and Picard tools (https://github.com/broadinstitute/picard; accessed on 1 December 2024). Genomic variants (SNPs and small InDels) were jointly called across all samples using the GATK (v4.6.1.0) HaplotypeCaller pipeline following best practices [44]. A stringent filter was applied to obtain a high-confidence variant set, retaining only biallelic sites with QUAL ≥ 30, QD ≥ 2.0, MQ ≥ 40, and FS ≤ 60.0.

### 4.4. BSA and Identification of Candidate Regions

To map genomic regions associated with the node number trait, high-confidence SNPs and InDels were further filtered to retain informative sites showing polymorphism between the parents and adequate coverage (depth ≥ 4) in the bulks. Two complementary algorithms were employed for association analysis:

(1) Euclidean Distance (ED): The ED value, representing the allele frequency difference between the two bulks, was calculated for each variant. A sliding window approach was used to smooth the ED profile across chromosomes. Candidate intervals were defined where the smoothed ED value exceeded a genome-wide threshold (median + 3 standard deviations).
(2) Δ(SNP/InDel-index): The SNP-index (or InDel-index) for each bulk was calculated relative to the reference parent allele frequency. The Δ-index, representing the absolute difference in index values between the two bulks, was computed. A 95% confidence threshold, established via a permutation test (1000 iterations), was used to define significant genomic intervals.

The final candidate regions were determined by intersecting the significant intervals identified by both the ED and Δ-index methods for SNPs and InDels, respectively.

### 4.5. Annotation and Prioritization of Candidate Genes

Genomic coordinates of the final candidate regions were intersected with the soybean gene annotation file of assembly Wm82.a4.v1. Genes located within these regions were extracted as candidate genes. Within the candidate regions, variants were functionally annotated using SnpEff (v4.3t) to predict their effects [45]. Priority was given to genes harboring high-impact variants, such as those causing nonsynonymous substitutions or frameshifts. GO terms and KEGG pathways were assigned to the high-confidence candidate gene sets.

### 4.6. Phytohormone Profiling and Data Analysis

Fresh samples from two lines (LSD914 and HN48, six plants total) were ground in liquid nitrogen and extracted with solvent containing internal standards. After centrifugation, evaporation, and filtration, the extracts were analyzed using a UPLC-ESI-QTRAP 6500+ system (Sciex) coupled with a Waters HSS T3 C18 column. Data acquisition was performed in scheduled multiple reaction monitoring (MRM) mode. Metabolites were quantified via internal standard calibration. Differential hormones between lines were identified using thresholds of fold change ≥ 2 or ≤0.5. Statistical analyses and visualizations (e.g., heatmaps, KEGG enrichment) were conducted with R software (v4.4.4).

### 4.7. Transcriptome Sequencing and Assembly

Leaf and stem tissues were collected from HN48 and LSD914 at three key developmental stages, including V2 (second trifoliate), R2 (full flowering), and R4 (full pod). Three biological replicates were collected per tissue per stage. Total RNA was extracted and sequenced on an Illumina HiSeq4000 platform.

RNA-seq raw reads from 36 samples were then subjected to quality control. Raw sequencing data were processed using fastp (version 0.23.2) [41] with default parameters to remove adapter sequences, trim low-quality bases (quality score < 20), and filter out reads shorter than 50 bp.

The clean reads were aligned to the soybean reference genome (Wm82.a4.v1) using STAR (version 2.7.10a) with default parameters [46]. Transcriptome assembly was performed using StringTie (version 2.2.1) with default parameters [47] and employing the reference annotation to guide assembly.

### 4.8. Generation of a Reference Transcriptome and Annotation

To construct a comprehensive and non-redundant reference transcriptome, all assembled transcripts from individual samples were merged using the RTDmaker tool with default settings (https://github.com/anonconda/RTDmaker; accessed on 1 December 2024). This process integrates transcript models across samples and generates a unified set of transcripts (reference transcript dataset, RTD) that captures both annotated and novel isoforms.

The functions of gene and transcript, derived from the RTDmaker workflow, were annotated using multiple databases. Protein sequences predicted from the reference genome were aligned against the UniProtKB/Swiss-Prot database using DIAMOND (e-value ≤ 1 × 10^−5^) [48] to obtain high-confidence functional descriptions. Gene Ontology (GO) categories and Kyoto Encyclopedia of Genes and Genomes (KEGG) pathway annotation were performed using EggNOG-mapper [49] integrated with InterProScan (version 5.56-89.0) [50] to combine homology-based and domain-based evidence.

### 4.9. Expression Quantification and Differential Analysis

The abundance of transcripts in each sample was quantified using Salmon (version 1.9.0) in mapping-based mode [51]. An index was built from the RTD-derived transcript sequences, and quantification was performed with default parameters. The resulting transcript-level counts of transcripts per million (TPM) values were used for downstream analyses.

Differential expression analysis was conducted at both gene and transcript levels. For each pairwise comparison (D914 vs. HN48 within each tissue-stage combination), transcript-level counts from Salmon were summarized to gene-level expression quantities. Differential expression was assessed using the R package “DESeq2” (version 1.36.0) [52]. Significantly differentially expressed genes (DEGs) and transcripts (DETs) were identified based on an adjusted *p*-value (false discovery rate, FDR) < 0.01 and an absolute log2 fold change ≥1.

### 4.10. Functional Analysis and Overlap with BSA-Seq Candidates

To uncover biological functions and pathways associated with the identified DEGs, GO and KEGG enrichment analyses were performed separately for each pairwise comparison. Enrichment was tested using the hypergeometric test implemented in the “clusterProfiler” R package (version 4.4.4) [53]. GO terms and KEGG pathways with an adjusted *p*-value (FDR) < 0.05 were considered significantly enriched. The annotation mappings for GO and KEGG were derived from the functional annotation results described above.

Furthermore, we also analyzed the DEGs and DATs overlapping the candidate genes resulting from the BSA-seq analysis as aforementioned. The candidate gene supported by the expression results was annotated using both GO terms and KEGG pathways, and was also assigned by the functional descriptions for further investigations.

## 5. Conclusions

This study successfully employed an integrated multi-omics approach to delineate the genetic basis of increased main stem node number in soybean. We identified *GmGASA32* as a central regulatory gene, whose down-regulation, in concert with a unique phytohormone profile, appears to orchestrate a shift from internode elongation to node initiation. These findings provide a novel mechanistic insight into plant architecture regulation and offer valuable molecular targets for breeding high-yield soybean cultivars.

## Data Availability

The raw data generated in this study are available in the National Genomics Data Center (NGDC) under the BioProject PRJCA062458. The phytohormone profiling data have been supplemented.

## Supplementary Materials

The following supporting information can be downloaded at: https://www.mdpi.com/article/10.3390/plants15101418/s1. Figure S1. BSA-seq-based identification of candidate genomic regions associated with MSN using SNP and InDel markers; Figure S2. Annotation of BSA-seq identified variants (SNPs and InDels); Figure S3. Genome-wide identification of candidate genomic regions associated with MSN number using two analytical methods and InDel variants; Figure S4. Volcano plots showing differential expression at the gene and transcript levels of leaf between LSD914 and HN48; Figure S5. Volcano plots showing differential expression at the gene and transcript levels of the root between LSD914 and HN48. Supplementary Table S1. Statistics of the BSA-seq dataset; Supplementary Table S2. Statistics of variations and their annotations; Supplementary Table S3. Information on SNPs within the candidate regions, Supplementary Table S4. Information on InDels within the candidate regions; Supplementary Table S5. List of final candidate regions from BSA-seq analyses; Supplementary Table S6. List of genes within the BSA-derived candidate regions; Supplementary Table S7. Information on BSA-derived genes with nonsynonymous SNPs; Supplementary Table S8. Information on BSA-derived genes with frameshift InDels; Supplementary Table S9. GO enrichments of the candidate genes with non-synonymous SNPs or frameshift InDels; Supplementary Table S10. KEGG pathways of the candidate genes with non-synonymous SNPs or frameshift InDels; Supplementary Table S11. Information on phytohormone profilings; Supplementary Table S12. Phytohormones of significant differential accumulation between HN48 and LSD914; Supplementary Table S13. Numbers of differentially expressed genes and transcripts; Supplementary Table S14. Overlaps of DEGs and BSA-derived genes; Supplementary Table S15. Overlaps of DETs and BSA-derived genes; Supplementary Table S16. QTLs for the *GmGASA32* genes between HN48 and LSD419.

## Abbreviations

The following abbreviations are used in this manuscript:

GO	Gene Ontology
KEGG	Kyoto Encyclopedia of Genes and Genomes
DEG	Differentially expressed gene
DET	Differentially expressed transcript
TPM	transcripts per million
SNP	Single-nucleotide polymorphism
ED	Euclidean Distance
cZ	cis-zeatin
