# Supplementary material for: Multi-Omics Integration Unravels the Genetic and Hormonal Regulatory Mechanisms Underlying Increased Main Stem Node Number in Soybean
Source: Plants (Basel). 2026 May 7;15(10):1418. doi: 10.3390/plants15101418 (PMC13211102; doi:10.3390/plants15101418)
Supplement: Supplementary file 1 [file plants-15-01418-s001.zip › SupplementaryFigures.pdf]

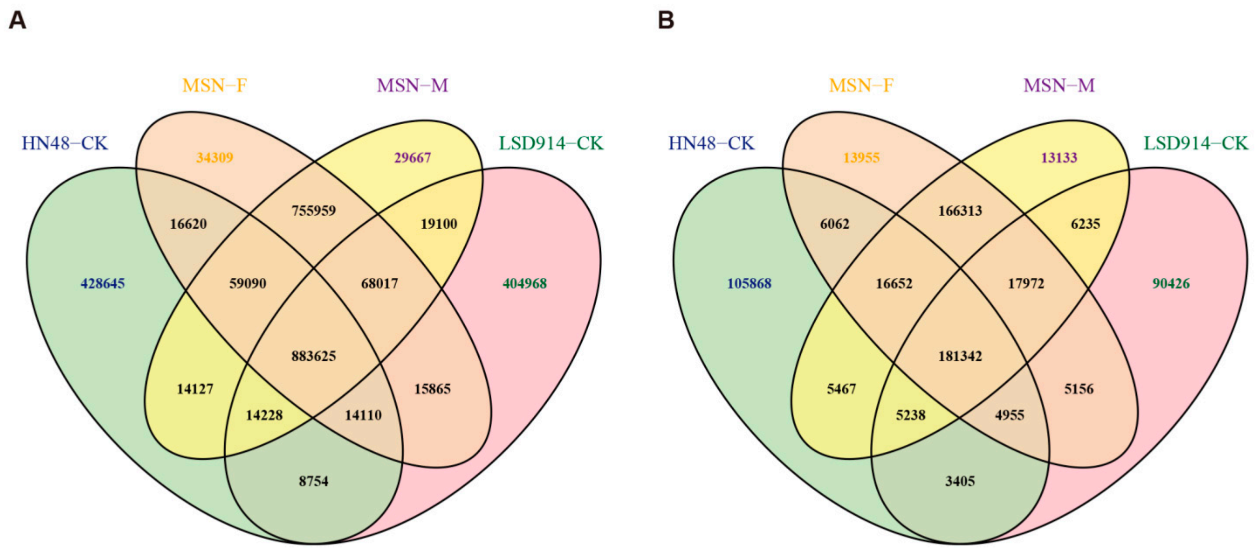

Figure S1. BSA-seq based identification of candidate genomic regions associated with MSN using SNP and InDel markers. (A) Genome-wide distribution of SNP variants. (B) Genome-wide distribution of InDel variants.

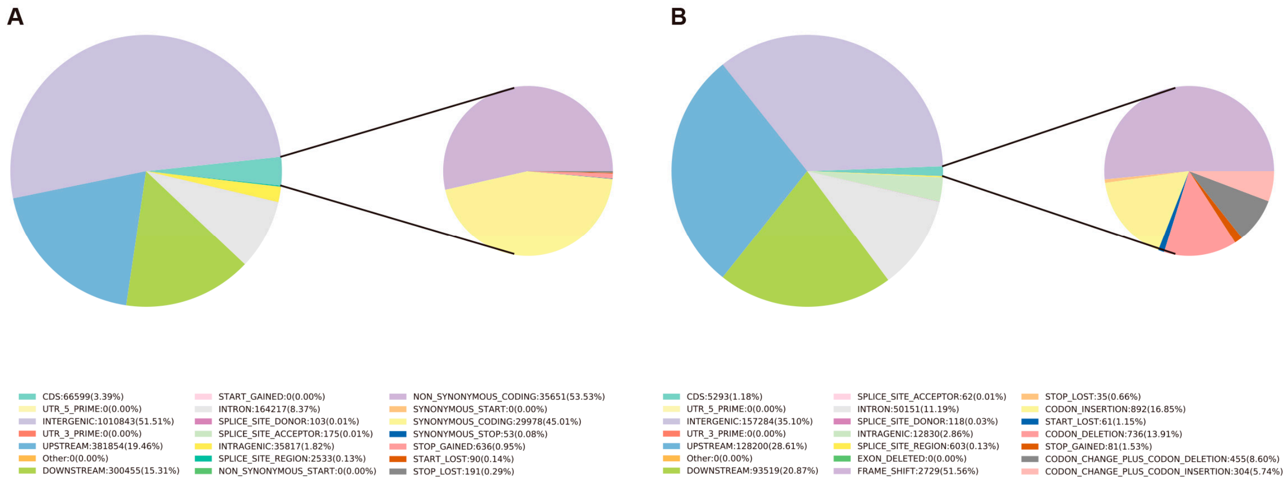

Figure S2. Annotation of BSA-seq identified variants (SNPs and InDels). (A) Functional annotation of SNP variants. (B) Functional annotation of InDel variants.

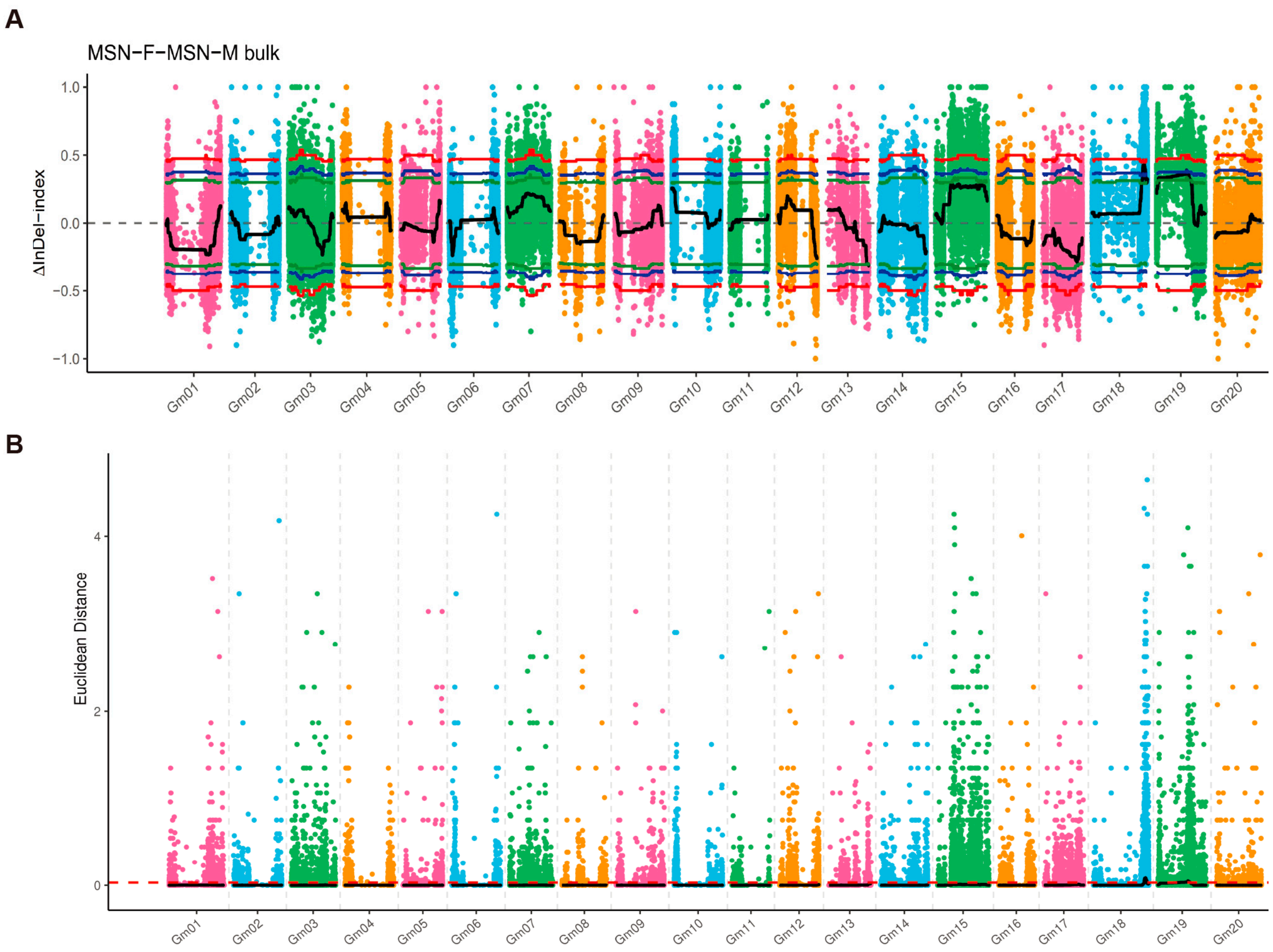

Figure S3. Genome-wide identification of candidate genomic regions associated with MSN number using two analytical methods and InDel variants. (A) Δ(InDel-index) analysis of InDels across the soybean genome.The plot displays the Δ(InDel-index) value for each InDel, with candidate regions highlighted above the significance threshold. (B) Euclidean distance (ED) analysis based on the same InDeldataset.

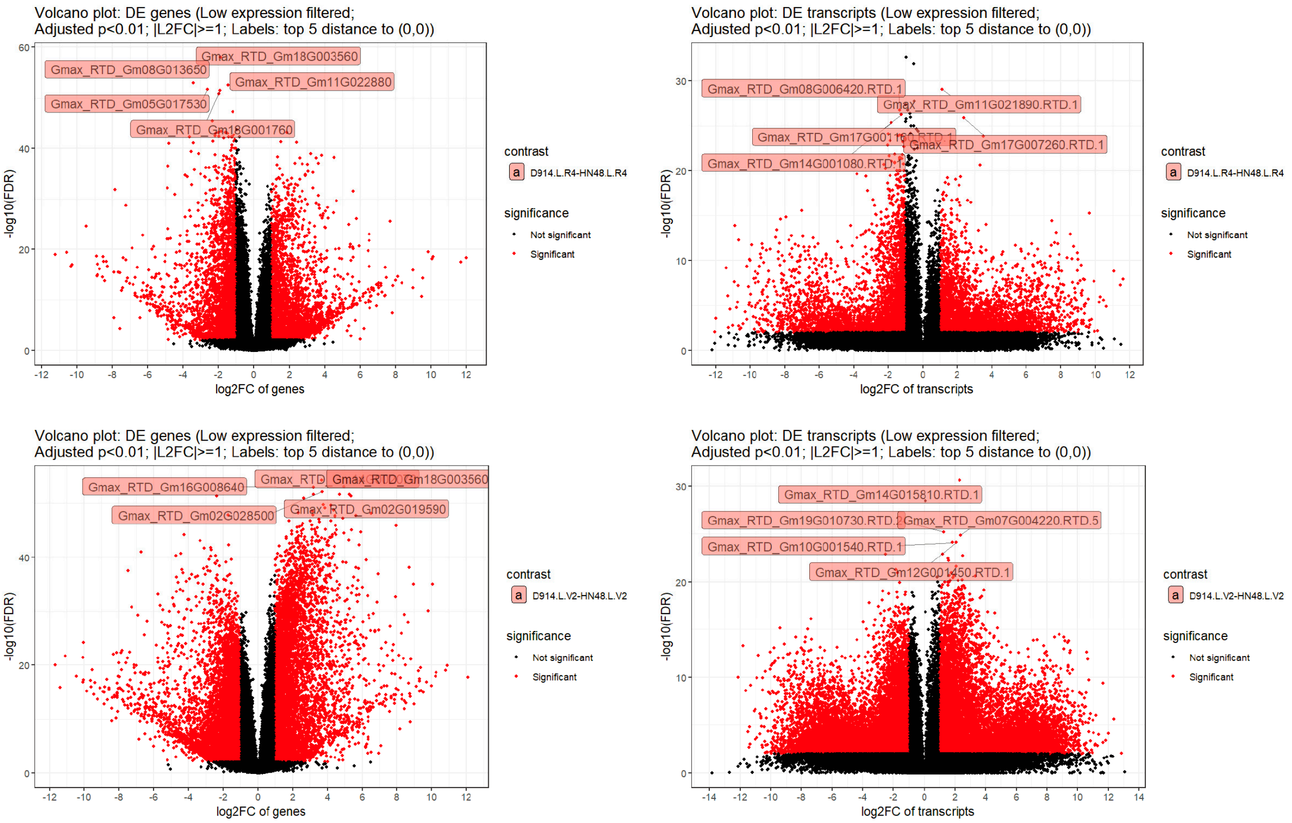

Figure S4. Volcano plots showing differential expression at the gene and transcript levels of leaf between LSD914 and HN48.

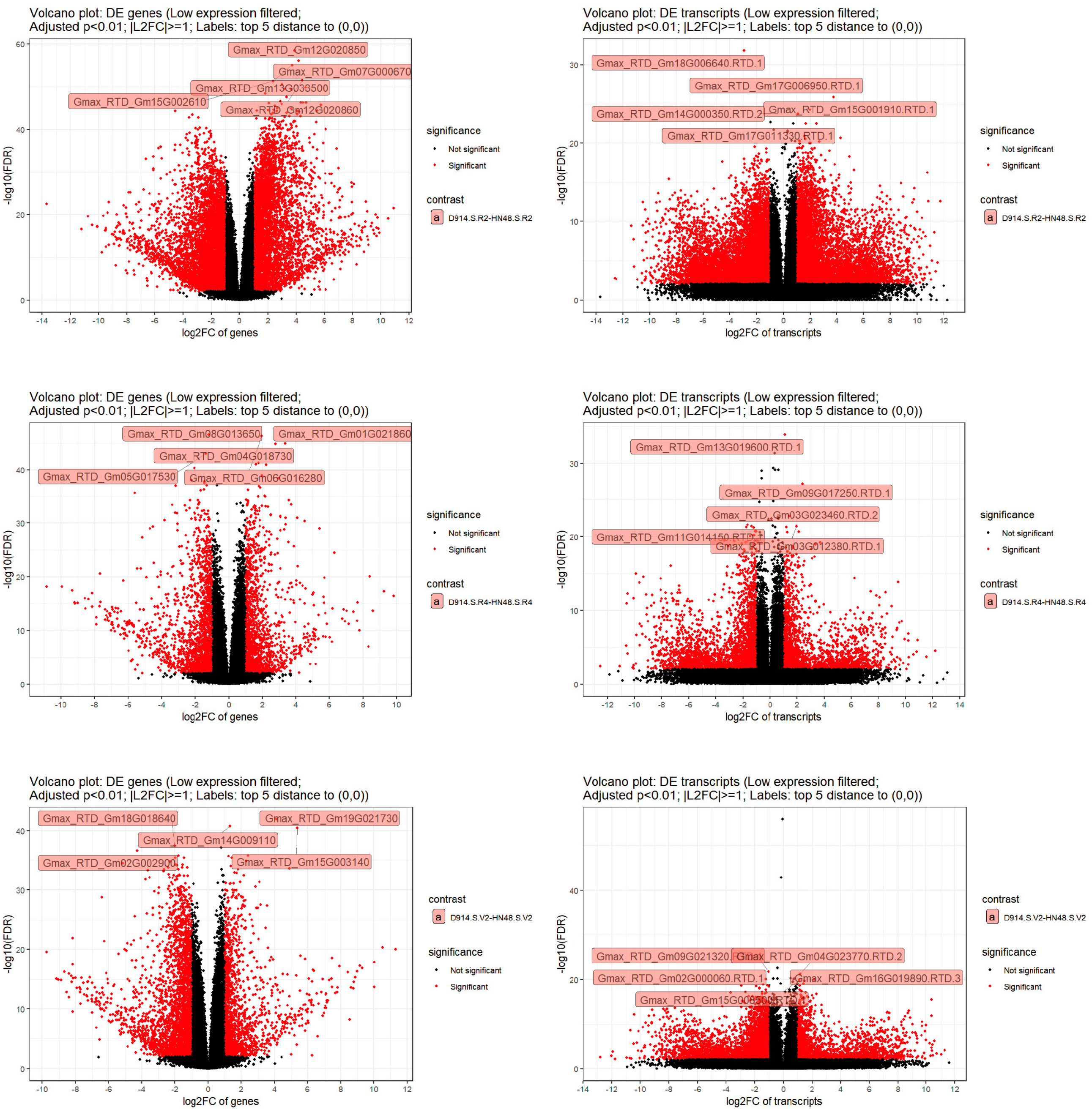

Figure S5. Volcano plots showing differential expression at the gene and transcript levels of root between LSD914 and HN48.
